# 2508. Evaluation of Infectious Diseases Complications in US Veterans with Opioid Use Disorder, a Single Site Experience

**DOI:** 10.1093/ofid/ofad500.2126

**Published:** 2023-11-27

**Authors:** Pronoma Srivastava, Viraj Modi, Audun J Lier

**Affiliations:** Stony Brook University Hospital, Stony Brook, NY; Northport VA Medical Center, Northport, New York; Northport VA Medical Center, Northport, New York

## Abstract

**Background:**

Opioid use disorder (OUD) affects 2.7 million people in the United States and rates of OUD diagnosis are rising in US Veterans (USV). Injection drug use (IDU), including opioids, can lead to acquisition of bloodborne infections and severe injection related infections (SIRI). This study aims to describe infectious diseases epidemiology, substance use characteristics, and health care utilization in persons who inject drugs (PWID) and non-PWID with OUD who presented to the Northport Veterans Affairs Medical Center (NVAMC).

**Table 1.**
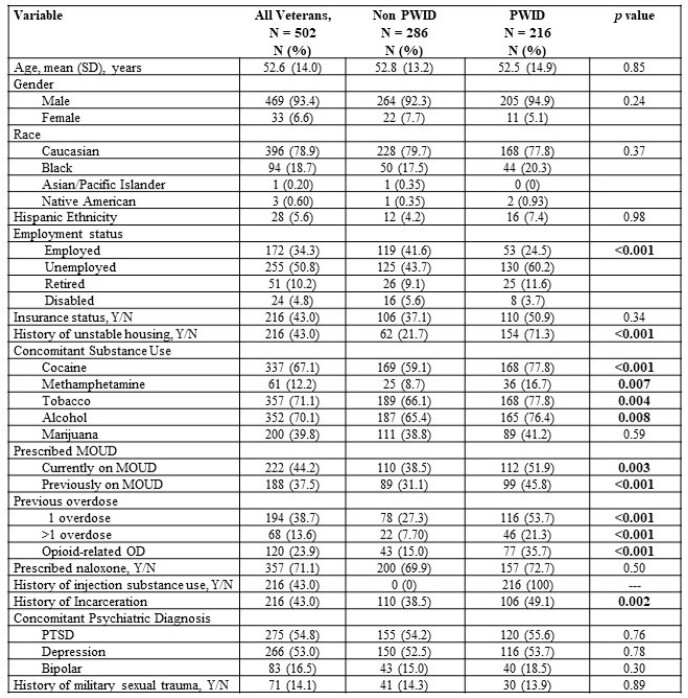
Demographic characteristics of USV with OUD, stratified by history of injection substance use. OUD, opioid use disorder. MOUD, medication for opioid use disorder. USV, US Veterans. PTSD, post-traumatic stress disorder.

**Methods:**

Data was collected from a retrospective chart review of Veterans aged >18 years who had an ICD9 or ICD10 diagnosis of OUD and presented to the NVAMC between 2010-2020. Demographics, concomitant substance use and prior overdose histories, receipt of medications for opioid use disorder (MOUD), prior infection histories, hospitalizations, and emergency department (ED) visits were compared between PWID and non-PWID. Two-sample T-tests and Chi-square analyses were utilized.

**Results:**

We identified 502 USV with a diagnosis of OUD. Mean age was 52.6 years, 469 (93.4%) were male, 396 (78.9%) were white, 172 (34.3%) were employed and 216 (43%) had health insurance. Post-traumatic stress disorder (n=275, 54.8%) was the most frequent psychiatric diagnosis and 216 (43%) USV had a history of IDU. PWID were more likely to have unstable housing (71.3% PWID vs 21.7% non-PWID, p< 0.001), be unemployed (60.2% PWID vs 43.7% non-PWID, p< 0.001), use cocaine (77.8% PWID vs 59.1% non PWID, p< 0.001), have a history of incarceration (49.1% PWID vs 38.5% non PWID, p=0.002), and have a prior overdose (53.7% PWID vs 27.3% non PWID, p< 0.001). Among PWID, 134 (62%) had hepatitis C virus and 30 (13.9%) had at least 1 SIRI, most frequently a skin and soft tissue infection (n=29, 13.4%). PWID were more likely to be hospitalized with an infection (26.4% PWID vs. 12.2% non PWID, p< 0.001) and had more inpatient admissions (5.5 PWID vs. 3.51 non PWID, p=0.003). ED visit frequency did not differ between groups.

**Table 2.**
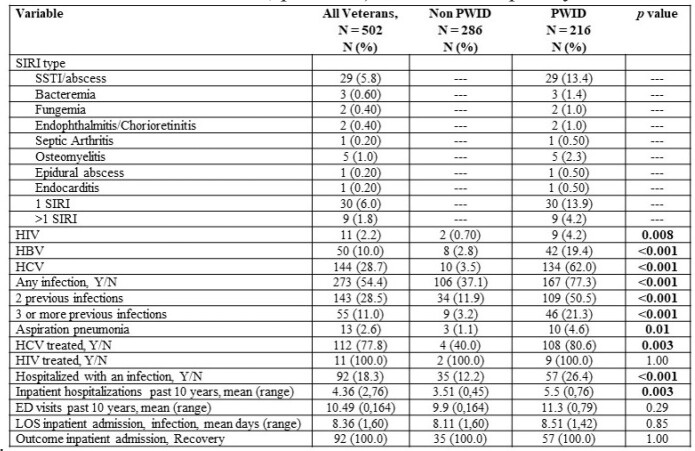
Characteristics of SIRI and non SIRI among Veterans with OUD presenting to the Northport VAMC. SIRI, severe injection-related infection. OUD, opioid use disorder. SSTI, skin and skin structure infection. HBV, hepatitis B virus. HCV, hepatitis C virus. LOS, length of stay.

**Conclusion:**

Veterans with OUD frequently present to the NVAMC with infections that require hospitalization. USV with OUD, especially PWID, would benefit from increased social support, as well as screening and treatment for OUD related infections.

**Disclosures:**

**All Authors**: No reported disclosures

